# Gene expression patterns in shoots of *Camelina sativa* with enhanced salinity tolerance provided by plant growth promoting bacteria producing 1-aminocyclopropane-1-carboxylate deaminase or expression of the corresponding *acdS* gene

**DOI:** 10.1038/s41598-021-83629-8

**Published:** 2021-02-19

**Authors:** Zohreh Heydarian, Margaret Gruber, Cathy Coutu, Bernard R. Glick, Dwayne D. Hegedus

**Affiliations:** 1 Agriculture and Agri-Food Canada, 107 Science Place, Saskatoon, SK S7N 0X2 Canada; 2 Department of Biology, University of Waterloo, Waterloo, ON Canada; 3 Department of Food and Bioproduct Sciences, University of Saskatchewan, Saskatoon, SK Canada; 4 Department of Biotechnology, School of Agriculture, University of Shiraz, Bajgah, Shiraz, Fars Iran

**Keywords:** Molecular biology, Plant sciences

## Abstract

Growth of plants in soil inoculated with plant growth promoting bacteria (PGPB) producing 1-aminocyclopropane-1-carboxylate (ACC) deaminase or expression of the corresponding *acdS* gene in transgenic lines reduces the decline in shoot length, shoot weight and photosynthetic capacity triggered by salt stress in *Camelina sativa*. Reducing the levels of ethylene attenuated the salt stress response as inferred from decreases in the expression of genes involved in development, senescence, chlorosis and leaf abscission that are highly induced by salt to levels that may otherwise have a negative effect on plant growth and productivity. Growing plants in soil treated with *Pseudomonas migulae* 8R6 negatively affected ethylene signaling, auxin and JA biosynthesis and signalling, but had a positive effect on the regulation of genes involved in GA signaling. In plants expressing *acdS*, the expression of the genes involved in auxin signalling was positively affected, while the expression of genes involved in cytokinin degradation and ethylene biosynthesis were negatively affected. Moreover, fine-tuning of ABA signaling appears to result from the application of ACC deaminase in response to salt treatment. Moderate expression of *acdS* under the control of the root specific *rolD* promoter or growing plants in soil treated with *P. migulae* 8R6 were more effective in reducing the expression of the genes involved in ethylene production and/or signaling than expression of *acdS* under the more active Cauliflower Mosaic Virus *35S* promoter.

## Introduction

The ability of *Camelina sativa* (camelina) to grow on marginal lands has piqued interest in its development as an industrial oilseed crop for biofuels, bio-lubricants and animal feed^1,2^. *C. sativa* is an allopolyploid that arose from a genome triplication event^3^ resulting in a 750 Mb genome encoding 89,418 proteins^4^. The genome remains highly undifferentiated with little fractionation bias; this presents significant challenges for breeding and genetic manipulation^4–6^, necessitating alternate strategies for trait improvement.

Accumulation of salts at or near the soil surface is a widespread agricultural problem that causes cellular dehydration and ion toxicity in plants^7,8^. These have deleterious effects on biochemical reactions resulting in plasmolysis, accumulation or reduction of specific secondary metabolites, nutrient imbalance, and production of reactive oxygen species, all of which can interfere with photosynthesis^9^. The consequences of these physiological changes are inhibition of seed germination, reduction in plant growth and vigor, changes to flowering time, and reduction in seed production^7,10^. While plants may respond somewhat differently to various stresses, nearly all respond by producing ethylene. Following the onset of stress, a small increase in ethylene production induces the transcription of defense/stress response genes. A subsequent and larger burst of ethylene is generated by persistent stress that acts as a signal to initiate processes, such as senescence, chlorosis and abscission, all of which inhibit plant growth^11,12^. The synthesis of stress ethylene can be reduced by lowering the level of the ethylene precursor, 1-aminocyclopropane-1-carboxylate (ACC), by applying the enzyme ACC deaminase either in the form of bacteria producing the enzyme or by introducing the bacterial *acdS* gene into the plant. These approaches significantly reduce the extent of inhibition on plant growth and development that normally accrues from stress^13–16^. Canola (*Brassica napus*) grown in soil treated with a strain of *Pseudomonas putida* producing ACC deaminase exhibited up-regulation of genes encoding auxin response factors and down-regulation of stress response genes^11^. Furthermore, transgenic *B. napus* expressing *acdS* performed better under salinity stress compared to non-transgenic plants^17^. Similarly, *C. sativa* lines expressing *acdS* or treated with bacteria producing ACC deaminase exhibited increased salinity tolerance^16^; this dramatically affected the transcriptional response in the roots of these plants under salt stress^18^. Here, we examined how expression of *acdS* in transgenic *C. sativa* under the control of broadly constitutive (CaMV *35S*) or root-specific (*rolD*) promoters, or treatment with plant growth promoting bacteria (PGPB) producing ACC deaminase affected the transcription of genes in vegetative tissues in response to salt stress.

## Materials and methods

### Bacterial strains

Two PGPB root endophytes that produce ACC deaminase, *Pseudomonas migulae* 8R6 and *Pseudomonas fluorescens* YsS6^19^, that were previously shown to increase salinity tolerance in *C. sativa*^16^ were used in this study. Two derived *acdS* mutant strains, 8R6M and YsS6M, constructed previously by insertion of a transposon containing the tetracycline resistance gene at position 237 in the *P. migulae* 8R6 *acdS* gene and at position 323 in the *P. fluorescens* YsS6 *acdS* gene^13^ were also examined. The ACC deaminase activity for these bacteria was determined previously^13^. The ACC deaminase activity of the wild-type *P. fluorescens* YsS6 was 12.5 mmol mg^−1^ h^−1^, while the activity of the corresponding *acdS* mutant was 0.11 mmol mg^−1^ h^−1^. The ACC deaminase activity of the wild-type *P. migulae* 8R6 was 10.9 mmol mg^−1^ h^−1^ and the activity of the corresponding *acdS* mutant was 0.03 mmol mg^−1^ h^−1^.

### Transgenic ***C. sativa*** lines expressing acdS

*Camelina sativa* cv. DH55 lines expressing the *Pseudomonas* sp. UW4^20^ *acdS* gene under the control of either the double cauliflower mosaic virus (CaMV) *35S* promoter or the *rolD* promoter from *Agrobacterium rhizogenes* were constructed previously^16^. The *P*. sp. UW4 ACC deaminase is 99% identical (amino acid level) to that from *P. fluorescens* YsS6 and 98% identical to that from *P. migulae* 8R6^16^. Individual, single copy lines in which the expression of the *P.* sp. UW4 *acdS* gene was verified using droplet digital PCR^16^ and RNA-Seq data^18^ were used.

### PGPB and salt treatment

Bacteria were cultured for 24 h in tryptic soy broth (TSB) containing 100 μg ml^−1^ ampicillin for wild-type strains or 100 μg ml^−1^ ampicillin and 10 μg ml^−1^ tetracycline for the *acdS* mutant strains^21^. Bacterial cultures were centrifuged at 4000×*g* and washed three times with 0.03 M MgSO_4_ prior to re-suspending to an OD_600nm_ = 0.50 ± 0.02 in 0.03 M MgSO_4_ (approximately 5.0 × 10^8^ bacteria/ml). Soil was inoculated with either 2 ml of PGPB or 2 ml of 0.03 M MgSO_4_ (control) at the time of sowing and again 1 week after sowing.

*Camelina sativa* cv. DH55 seeds were sown in soil-less potting mixture^22^ in 10 × 10 × 8 cm square pots. All materials were available locally, except fritted trace elements (Frit Industries Inc., Ozark, USA). NaCl solutions at 192 and 213 mM were prepared to obtain solutions with electrical conductivities (EC) of 15 dS m^−1^ and EC 20 dS m^−1^ at 20 °C, respectively. Tap water (50 ml; EC 248 µS m^−1^) was applied daily to each pot and replaced with 50 ml of saline solution 20 days after sowing. The accumulation of salt in the pots was controlled by draining and the EC of the drained water was measured weekly. Plants were grown in a controlled growth cabinet (16 h light/8 h dark; 20/17 °C) supplemented with halogen lights. Shoot material was harvested 20 days after the initial salt treatment just as plants began to flower, at which time the length and dry weights were recorded. Shoot length was measured from axis of the first true leaf petiole with the stem to the tip of the shoot and recorded to the nearest millimeter. For dry weight, samples were placed in pre-weighed, dry beakers and then dried at 80 °C for 48 h in a drying oven and weight recorded to the nearest milligram.

### Element, chlorophyll and ethylene measurement

The quantity of elements, chlorophyll and ethylene were determined in shoots of 33-day-old plants; 13 days after initial salt treatment and before plants exhibited severe symptoms of salt stress^18^. Samples were dried at 80 °C for 48 h and ground using liquid nitrogen. Sulphur (S), copper (Cu), boron (B), calcium (Ca), iron (Fe), potassium (K), magnesium (Mg), manganese (Mn), sodium (Na), phosphorus (P), and zinc (Zn) content were determined by Agvise Laboratories Inc. (Benson, MN, USA). The method employed involved digestion with nitric acid/hydrogen peroxide and determination of elements using inductively coupled plasma mass spectrometry on a Perkin Elmer 5400 analyzer (Perkin Elmer, Guelph, Canada) according to Wu et al.^23^.

Chlorophyll content was measured using a chlorophyll meter (Apogee Instruments Inc., Logan, UT, USA) and photosynthesis yield (Fv/Fm) using a FV/FM meter (ADC Bioscientific Ltd., UK)^12^.

For ethylene measurement, 4 g (wet weight) of vegetative tissue was placed in 40 ml glass vials. The vials were sealed with rubber stoppers and placed at 20 °C for 6 h. 20 ml of gas was removed with a syringe and injected into a pre-evacuated Exetainer soda glass vial paired with a cap constructed with both a PTFE/silicone and a chlorobutyl septum (Labco Ltd., Ceredigion, UK). Ethylene was separated by gas chromatography (Bruker 450 GC, Bruker Biosciences Corporation, USA) and its concentration was calculated by comparing the peak area and peak length to standard peaks generated with pre-determined ethylene concentrations. Ethylene production was reported as parts per million ethylene/g fresh weight/hour. The measurements were repeated with three independent biological replicates.

### RNA sequencing and data analysis

Total RNA was extracted from shoot tissue of three biological replicates for each of the control, *35S::acdS*, *rolD::acdS* and *P. migulae* 8R6 treated lines (12 samples in total) from 28-day-old plants (21 days after 15 dS m^−1^ salt treatment began and before plants started to bolt) using an RNeasy Plant Mini Kit (Qiagen Inc.)^12,18^. The cDNA libraries were prepared using the TruSeq Stranded mRNA and Total RNA Library Prep kits with TruSeq LT adaptors (Illumina Inc.). The tagged libraries were sequenced using a HiSeq 2500 (Illumina Inc.)^12,18^.

The short-read sequence data from the 12 libraries were deposited in the NCBI GEO database (GSE132600). Trimmomatic^24^ was used to discard low-quality reads, to remove adaptor sequences and low quality nucleotides at the beginning or end of the read (PHRED33 quality score of less than 3), and to discard short reads (under 21 nt). The retained high-quality reads were mapped to the *C. sativa* reference genome using STAR RNA-seq aligner^25^ and transcripts available in the NCBI *C. sativa* release 100 (JFZQ00000000.1^4^). Expression levels for each gene were measured as counts^26,27^. Normalization for library size was performed for each gene by dividing the counts by the library size as described^28^ to yield counts per million (CPM). Further normalization was performed using DESeq to approximate a negative binomial distribution. The differential expression analysis of digital gene expression data software (edgeR) was used to calculate differences in expression between libraries^26^. The Biological Coefficient of Variation (BCV) value was set to 2 according to the software's instructions. Expression changes were declared to be significant if the multiple test corrected *p* value and the false discovery rate (FDR) were ≤ 0.05 and the absolute value of log 2 CPM was higher than 1. MAPMAN software version 3.5.1R2 (http://mapman.gabipd.org/) was used to assign gene ontology (GO) terms to unigenes based on molecular function, biological processes and cellular compartment^29^. Cluster analysis was done using JMP software version 15 (https://www.jmp.com/en_ca/home.html).

### Quantitative droplet digital PCR (ddPCR) analysis

Quantitative ddPCR was used to confirm the expression profiles of a set of genes as determined by the RNA-Seq analysis. Total RNA was extracted from shoots of 3 independent biological replicates (control, *35S::acdS*, *rolD::acdS* and *P. migulae* 8R6 treatment) using the RNeasy Plant Mini kit (Qiagen) and cDNA synthesized using the SuperScript III First-Strand Synthesis kit (Thermo Fisher Scientific). One ng of cDNA was used as template in each PCR reaction mixture with 2× Supermix (Bio-Rad Laboratories Inc.), 500 nM of each primer and 250 nM of each probe in a final volume of 20 µl. The sequences of the primers and probes are provided in Supplemental Data 1. Data were analyzed using Quanta-Soft version 1.7.4.0917 (Bio-Rad Laboratories Inc.) and the relative ratio of the candidate gene expression was calculated relative to the expression of the *actin* reference gene by plotting the concentration of FAM- over the HEX-labelled probe according to the Bio-Rad ddPCR guide^12,18^.

### Statistical analysis

Plant growth measurements were expressed as the mean ± standard error for each treatment. Significant differences between treatments were determined by variance analysis (ANOVA) with a *p* value of ≤ 0.05 and pair-wise comparisons were conducted using the Tukey's Studentized Range (HSD) test using SAS 9.3 (TS1M2)^12,16,18^.

## Results

### Ethylene, plant growth, photosynthetic capacity, and element composition

**Figure 1:**
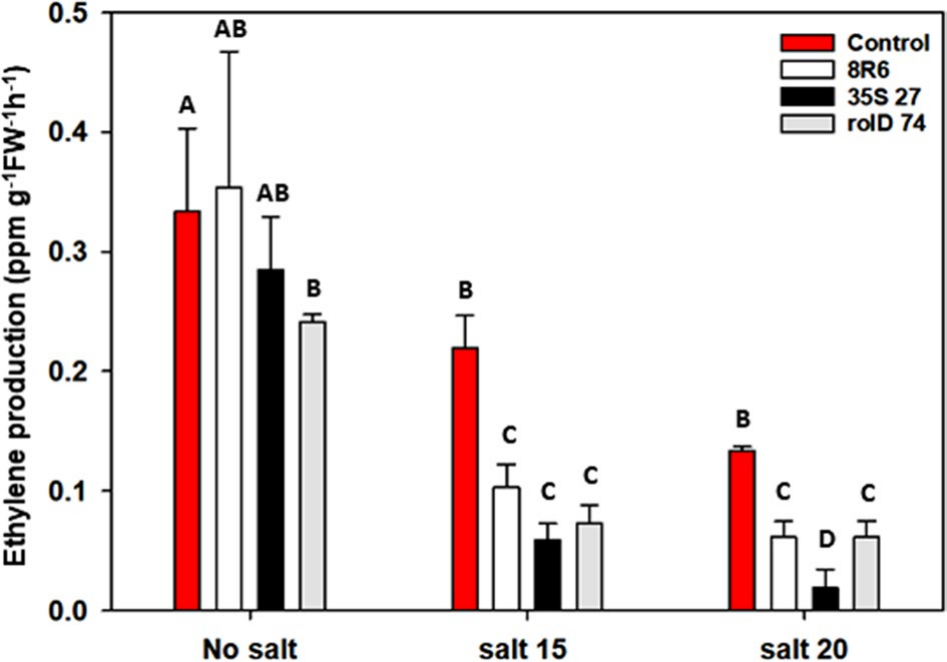
The effect of ACC deaminase on ethylene production in *C. sativa* under salt stress (NaCl; 15 and 20 dS m^−1^). Salt was applied 20 days after sowing and plant material collected 13 days afterward. Soil was treated with buffer (control) or the *P. migulae* 8R6 strain producing ACC deaminase. Transgenic lines tested were independent, single insert, homozygous lines expressing the *Pseudomonas* sp. UW4 *acdS* gene under the control of either the *rolD* or the CaMV *35S* promoter. Error bars indicate standard error (n = 3 biological replicates). Significant differences between groups were detected using a two-way ANOVA and Tukey post-test. Values that are significantly different (p < 0.05) are indicated by letters. Plots were drawn using SigmaPlot ver. 13.0 (Systat Software, Inc., San Jose, USA, www.systatsoftware.com).

Expression of *acdS* or treatment of soil with *P. migulae* 8R6 had only a marginal effect on the levels of ethylene in vegetative tissue in the absence of salt (Fig. 1). Overall, levels of ethylene were lower in all lines sampled at 13 days after the application of salt; however, the levels were significantly decreased by the presence of ACC deaminase, either by applying *P. migulae* 8R6 to the soil or via *acdS* expression in the transgenic lines, at both moderate and high salt concentrations (EC 15 and 20 dS m^−1^). The line expressing *acdS* under the control of the CaMV *35S* promoter had the lowest levels of ethylene at the highest salt concentration.

**Figure 2:**
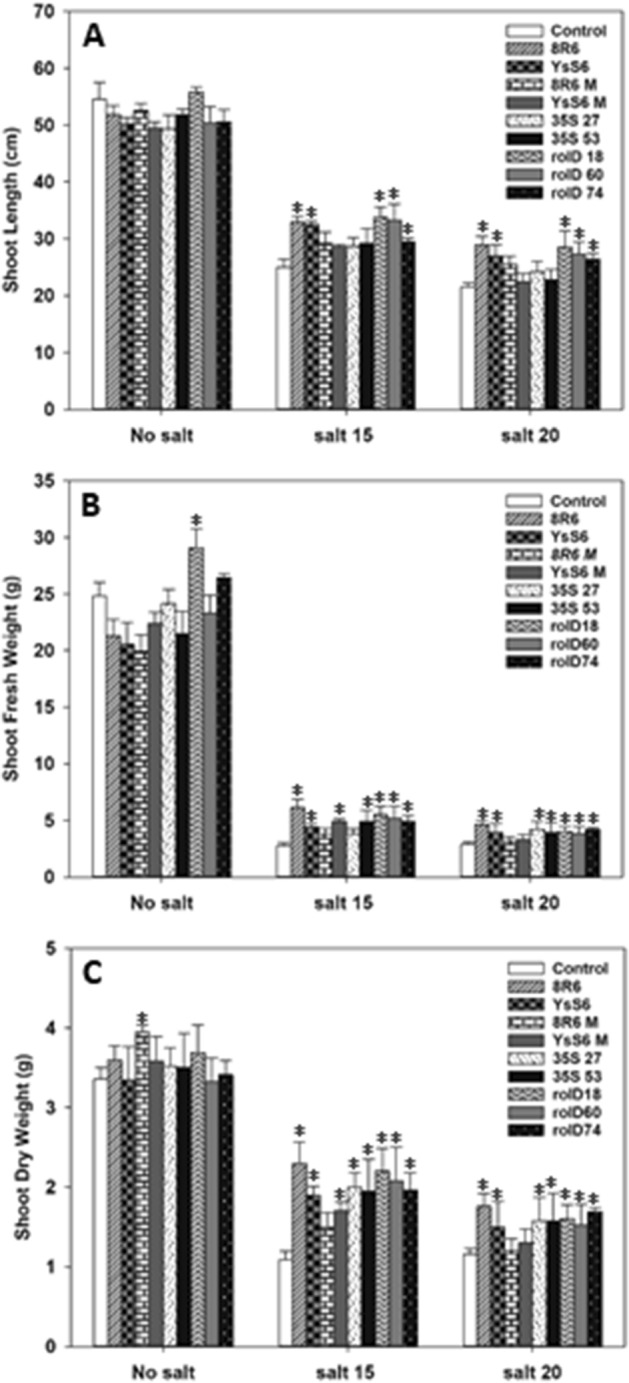
The effect of ACC deaminase on *C. sativa* shoot length (**A**), fresh weight (**B**), and dry weight (**C**) in the absence or presence of salt stress (NaCl; 15 and 20 dS m^−1^). Soil was treated with buffer (control), *P. migulae* 8R6 producing ACC deaminase or a derived *acdS* mutant 8R6 M, *P. fluorescens* YsS6 producing ACC deaminase or a derived *acdS* mutant YsS6 M. Transgenic lines tested were independent, single insert, homozygous lines expressing the *Pseudomonas* sp. UW4 *acdS* gene under the control of either the *rolD* or the CaMV *35S* promoter. Salt was applied 20 days after sowing and shoots were harvested 20 days afterward. Error bars indicate standard error (n = 10 biological replicates). Significant differences between groups were detected using a two-way ANOVA and Tukey post-test. Values that are significantly different (p < 0.05) from the control are indicated by asterisks (*) above the bars. Plots were drawn using SigmaPlot ver. 13.0 (Systat Software, Inc., San Jose, USA, www.systatsoftware.com).

As expected, shoot length, fresh and dry weight decreased as the salt concentration increased regardless of the treatment; however, the presence of ACC deaminase delivered either by PGPB or via *acdS* expression in transgenic lines significantly reduced the impact of salt stress (Fig. 2). The reduction in shoot length was significantly less for plants grown in soil treated with *P. migulae* 8R6 or *P. fluorescens* YsS6, but not with the corresponding *acdS*-mutants, as was also the case for transgenic lines expressing *acdS* under the control of the *rolD* promoter. Shoot fresh and dry weight responded similarly and the beneficial effect was also observed in lines expressing *acdS* under the control of the CaMV *35S* promoter. In addition, the control plants were severely wilted 20 days after treatment with the higher salt concentration (EC 20 dS m^−1^), whereas plants expressing *acdS* or treated with wild-type PGPB were not^18^.

**Figure 3:**
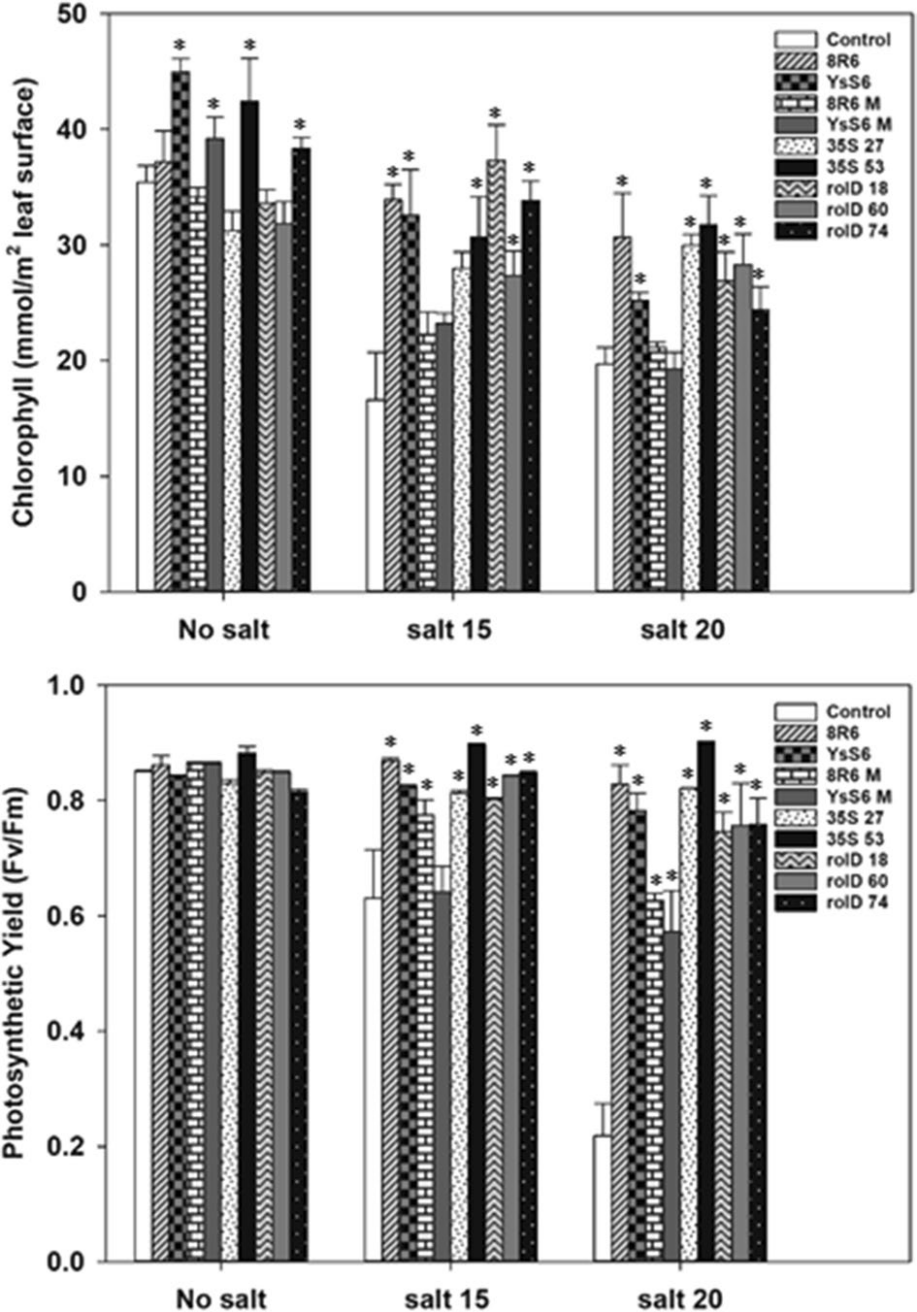
The effect of ACC deaminase on *C. sativa* photosynthetic capacity in the absence or presence of salt stress (NaCl; 15 and 20 dS m^−1^). Panels show chlorophyll content and photosynthetic yield 33 days after sowing with salt being applied 20 days after sowing. Soil was treated with buffer (control), *P. migulae* 8R6 producing ACC deaminase or a derived *acdS* mutant 8R6 M, *P. fluorescens* YsS6 producing ACC deaminase or a derived *acdS* mutant YsS6 M. Transgenic lines tested were independent, single insert, homozygous lines expressing the *Pseudomonas* sp. UW4 *acdS* gene under the control of either the *rolD* or the CaMV *35S* promoter. Error bars indicate standard error (n = 10 biological replicates). Significant differences between groups were detected using a two-way ANOVA and Tukey post-test. Values that are significantly different (p < 0.05) from the control are indicated by asterisks (*) above the bars. Plots were drawn using SigmaPlot ver. 13.0 (Systat Software, Inc., San Jose, USA, www.systatsoftware.com).

Chlorophyll content and photosynthetic yield (Fv/Fm) illustrate the level of activity in the photosystem reaction centers. Chlorophyll content was significantly higher in the *acdS* transgenic lines and in plants grown in soil treated with the wild-type PGPB under salt stress, but not with the *acdS*-mutants (Fig. 3). Photosynthetic yield was maintained to an even higher degree and was especially apparent at the higher salt concentration (EC 20 dS m^−1^). The effect was observed with all treatments, including the PGPB *acdS-*mutants as these bacteria possess additional mechanisms to promote plant growth and health^15,19^.

**Figure 4:**
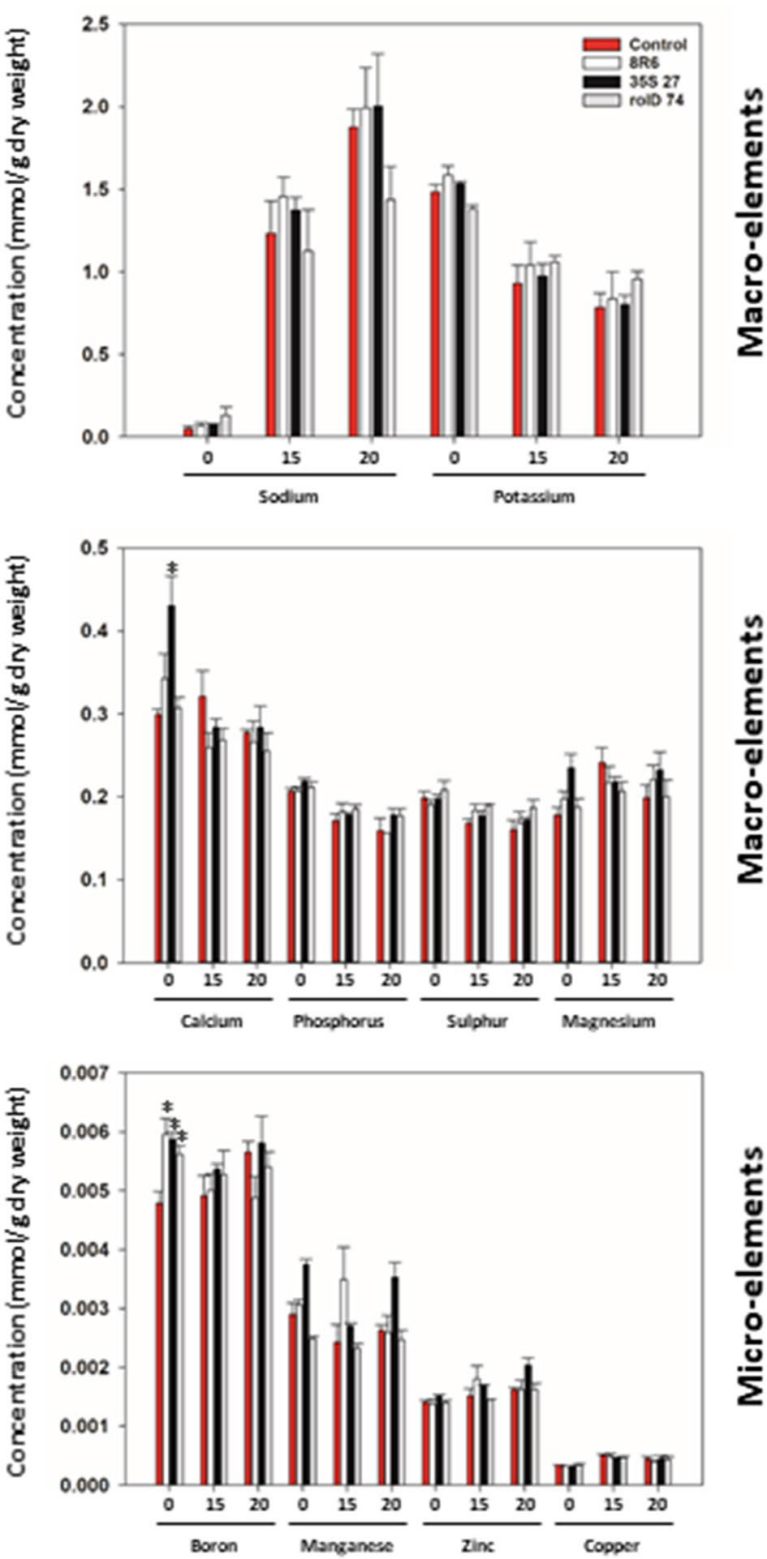
The effect of ACC deaminase on shoot element content in *C. sativa* grown in the absence or presence of salt. Soil was treated with buffer (control) or *P. migulae* 8R6 producing ACC deaminase. Transgenic lines tested were independent, single insert, homozygous lines expressing the *Pseudomonas* sp. UW4 *acdS* gene under the control of either the *rolD* or the CaMV *35S* promoter. Panels show macro- and micro-element content in shoots of plants 33 days after sowing that were irrigated with either tap water or with a salt solution (15 or 20 dS m^−1^); application of the salt solution began 20 days after sowing. Significant differences between groups were detected using a two-way ANOVA and Tukey post-test. Values that are significantly different (p < 0.05) from the control are indicated by asterisks (*). Error bars indicate standard error (n = 3 biological replicates). Plots were drawn using SigmaPlot ver. 13.0 (Systat Software, Inc., San Jose, USA, www.systatsoftware.com).

ACC deaminase had little effect on the concentration of macro- and micro-elements in vegetative tissues. The levels of some macro-elements changed with increasing salinity; for example, the sodium levels rose sharply and the potassium and calcium levels declined; however, expression of *acdS* or treatment of soil with *P. migulae* 8R6 had little or no impact on the levels or trends (Fig. 4). With respect to the micro-elements, the boron concentration was significantly elevated by *acdS* expression and the presence of *P. migulae* 8R6, but only in the absence of salt.

### Global changes in *C. sativa* transcription in response to salt treatment

ACC deaminase was demonstrated to improve the tolerance of *C. sativa* to salinity stress. To further explore this phenomenon, the transcriptome of the above ground (vegetative) tissue of the wild-type DH55 line was compared to tissues from lines expressing *acdS* under the control of the root-specific promoter (*rolD*) or the constitutive promoter (*CaMV 35S*), as well as to tissues from plants grown in soil treated with *P. migulae* 8R6. The *rolD* promoter was not entirely root-specific, since *acdS* expression in vegetative tissues was approximately one-half the level observed in roots. However, in vegetative tissues, the expression of the *acdS* gene was 60 times higher under the control of the CaMV *35S* promoter than under the *rolD* promoter (data not shown).

The gene expression patterns were defined in terms of the impact ACC deaminase had on the expression normally observed in the non-trangenic (wild-type) or untreated (not grown in soil amended with PGPB) control line under salt stress. These patterns include: “induced” or “down-regulated” when the expression of the gene was not affected by salt stress in the control, but was altered in the transgenic or PGPB-treated lines; “less induced” when the gene was induced in the control, but to a lesser degree in the transgenic or PGPB-treated lines and similarly for “less down-regulated”; “more induced” when the gene was induced in the control, but to a greater degree in the transgenic or PGPB-treated lines and similarly for “more down-regulated”.

**Figure 5:**
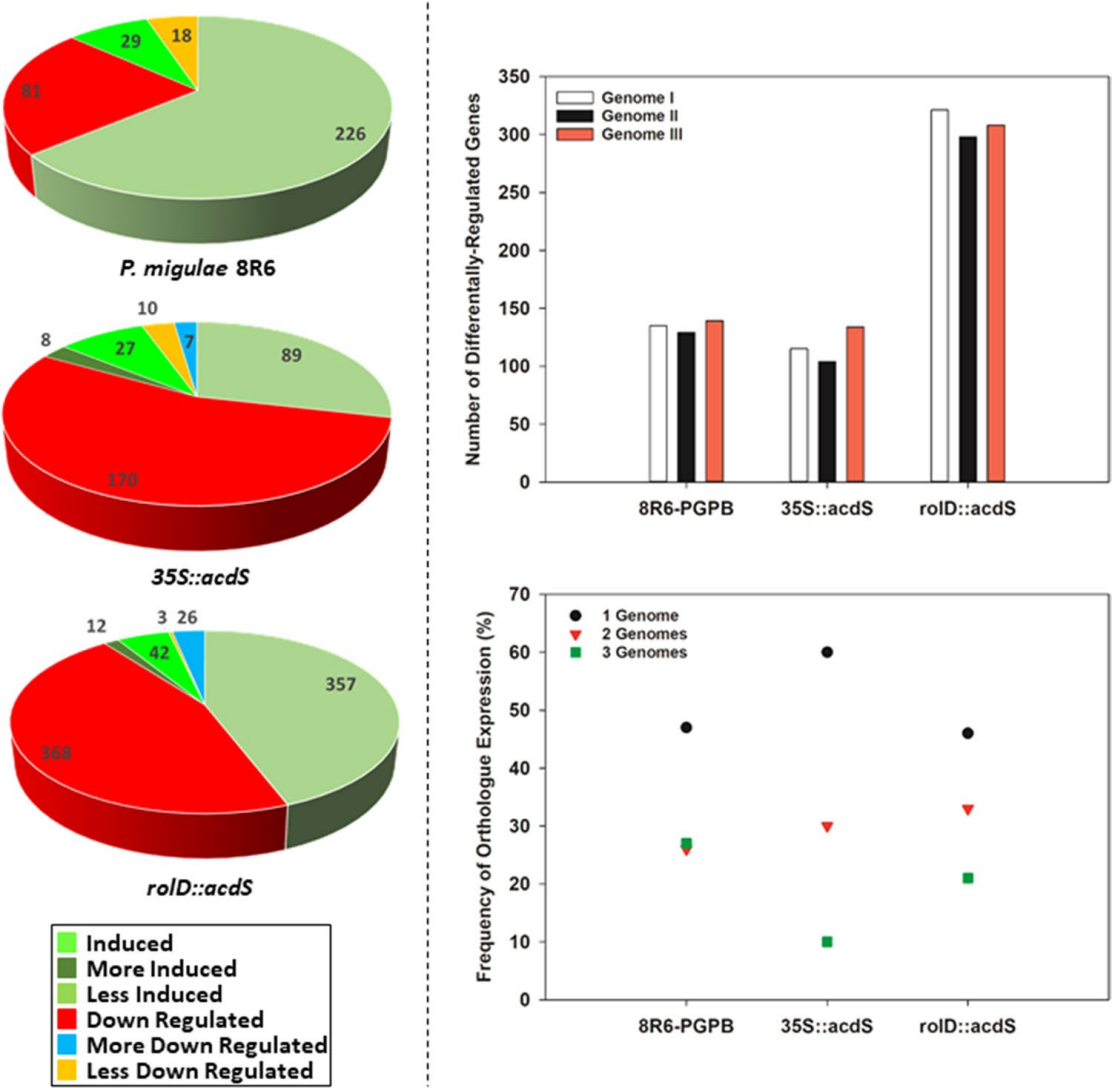
Differential-expression genes in vegetative tissue from *C. sativa* lines expressing *acdS* under the control of the *rolD* promoter or the constitutive CaMV *35S* promoter or from plants grown in soil treated with *P. migulae* 8R6 in the presence of salt (NaCl, 15 dS m^−1^). Left hand panel: Pie diagrams providing the number of genes that were differentially-regulated compared to the non-transgenic, untreated control. Right hand panel: plots representing the number of genes that were differentially-expressed on each of the *C. sativa* sub-genomes (top) and genome partitioning defined as the proportion of times when homeologous genes on one, two or three genomes were differentially-expressed (bottom). Genes with an FDR and p value ≤ 0.05 and with an absolute value of log 2 CPM higher than 1 were considered significant. Plots were drawn using SigmaPlot ver. 13.0 (Systat Software, Inc., San Jose, USA, www.systatsoftware.com).

In vegetative tissues of plants treated with *P. migulae* 8R6, 29 genes were induced and 81 genes were down-regulated, while 226 genes were less induced and 18 genes were less down-regulated compared to the control (DH55 not treated with *P. migulae* 8R6) in the presence of salt (FDR ≤ 0.05 and expression ≥ twofold; Fig. 5; Supplemental Data 2). In the *35S::acdS* line, 27 genes were induced, 170 genes were down-regulated, 8 genes were more induced, 89 genes were less induced, 7 genes were more down-regulated and 10 genes were less down-regulated. In the *rolD::acdS* line, 42 genes were induced, 368 genes were down-regulated, 12 genes were more induced, 357 genes were less induced, 26 genes were more down-regulated and 3 genes were less down-regulated compared to the control in the presence of salt. It is interesting to note that expression of *acdS* under the control of the *rolD* promoter had a much greater effect on the expression of salt-responsive genes than expression under the control of the much stronger CaMV *35S* promoter. In total, about 300 genes were differentially-regulated in the *35S::acdS* line, while the expression of about 800 genes was affected in the *rolD::acdS* line. Approximately, three times as many genes were less induced or more down-regulated in the *rolD::acdS* line compared to the *35S:*:*acdS* line or plants grown in soil treated with *P. migulae* 8R6 after exposure to salt.

Of the differentially-regulated genes in plants treated with *P. migulae* 8R6, approximately one third were assigned to each sub-genome with 121 assigned to Genome I, 110 to Genome II and 121 to Genome III. The fractional distribution in the *rolD::acdS* line was similar with 283 genes assigned to Genome I, 260 to Genome II and 284 to Genome III (Fig. 5). However in the *35S:*:*acdS* line, 120 genes were assigned to Genome III with only 101 assigned to Genome I and 89 to Genome II. An indication of partial genome partitioning in response to salt stress was found by examining the expression of homeologous genes. In plants grown in soil treated with *P. migulae* 8R6, 47% of the differentially-expressed genes were assigned to only one sub-genome, while 26% of the differentially-expressed genes (84 genes—42 homeologous pairs) were assigned to two sub-genomes, and 27% (87 genes—29 homeologous groups) were assigned to all three sub-genomes. In the *35S::acdS* line, 60% were assigned to only one sub-genome, while 30% (76 genes—38 homeologous pairs) were assigned to two sub-genomes, and only 10% (24 genes—8 homeologous groups) were assigned to all three sub-genomes. In the *rolD::acdS* line, 33% (212 genes—106 homeologous pairs) were assigned to two sub-genomes, whereas 21% (132 genes—44 homeologous groups) were assigned to all three sub-genomes with the remainder being assigned to only one sub-genome (46%) (Fig. 5).

To confirm the RNA-Seq data, a set of differentially-expressed genes was chosen for quantitative ddPCR analysis (Supplemental Information 3). The expression profiles of the genes were very similar regardless of the method. A complete annotation of the genes that were differentially-expressed in vegetative tissues of these lines can be found in Supplemental Data 4 and are discussed further below. Heat maps superimposed on MapMan GO pathways were used to visualize systems that were altered in response to salt stress and are available in Supplemental Information 4.

## Systems and biochemical pathways altered by ACC deaminase during response to salt

### Photosynthesis

Salt treatment negatively impacts photosynthesis and the expression of genes related to photosynthesis; however, ACC deaminase positively affected the expression of some genes involved in photosynthesis upon salt treatment (Supplemental Data 2). Csa08g050560 (photosystem I subunit D-1) was less down-regulated and three genes involved in the photosystem II light harvesting complex were less down-regulated (Csa14g037480 and Csa06g036870) or induced (Csa01g001010) in vegetative tissues of plants grown in soil treated with *P. migulae* 8R6 compared to the control DH55 line under salt stress. In the *35S::acdS* line, Csa17g025490 (chlorophyllase 1) was down-regulated compared to the wild-type DH55 line during salt stress.

### Cell wall biosynthesis/modification and secondary metabolism

The plant cell wall provides a structural framework for the mechanical and physical properties of the cell and determines the amount of water uptake and cell expansion available to support plant growth^30^. In the *rolD::acdS* line, which expressed *acdS* at low levels in vegetative tissues, cell wall-related genes were mostly down-regulated (40 genes down-regulated, 8 genes less induced, 10 genes more down-regulated and only 2 genes induced) in response to salt stress compared to wild-type DH55 (Supplemental Data 2). Interestingly, in the *35S::acdS* line, which has 60-fold more *acdS* transcripts in vegetative tissues than the *rolD::acdS* line, only 9 cell wall-related genes were differentially-regulated (3 genes down-regulated, 2 genes more down-regulated, 1 gene less down-regulated and 3 genes induced).

Secondary metabolites accumulate during salt stress, for example anthocyanins and flavonoids are generated in response to salinity stress and during leaf senescence in *A. thaliana*^31^. However, genes involved in the synthesis of secondary metabolites in plants grown in soil treated with *P. migulae* 8R6, or in the lines expressing *acdS* were generally less-induced or down-regulated (Supplemental Data 2).

### Phytohormones

Hormones are essential for plant growth and development, and are essential for adaptation to adverse biotic and abiotic environmental factors. ACC deaminase directly impacts the production of ethylene by limiting the amount of its immediate precursor. Interestingly, this was also found to indirectly affect the expression of genes involved in the synthesis, regulation or responses associated with other plant hormones as described below.

#### Auxin

Five genes related to auxin metabolism, including those encoding IAA-amino acid hydrolase ILR1-like 4 (Csa14g060060) and IAA-amino acid conjugate hydrolase ILR1-like 6 (Csa14g049310, Csa03g046940 and Csa17g070920) were less induced after salt treatment in the vegetative tissues of plants grown in soil treated with *P. migulae* 8R6. Five auxin-responsive genes encoding members of the Small Auxin Up RNA (SAUR) family, including Csa07g039520, Csa09g076010 and Csa16g035110 (SAUR 78), were induced or less down-regulated in the *35S::acdS* line, while Csa17g042390 (SAUR 66) was induced in the *rolD::acdS* line. In the *rolD::acdS* line, five auxin-responsive genes encoding GH3 family proteins were down-regulated (Csa16g044890, Csa09g087450, Csa07g053230, Csa08g005200) or less induced (Csa10g003770) (Supplemental Data 2).

#### Gibberellic acid (GA)

Csa01g002510 and Csa07g046090, which encode GA-Stimulated Arabidopsis (GASA) 5 and 6, respectively, were the only GA-dependent genes that were differentially-expressed. These were less down-regulated and only in plants grown in soil treated with *P. migulae* 8R6 (Supplemental Data 2).

#### Cytokinin

Cytokinin oxidase 3 (CKX3; Csa11g090460) catalyzes the degradation of cytokinins and was down-regulated fivefold in the *35S::acdS* line. Csa11g090460 and one of its homeologues (Csa02g061320) were less induced or down-regulated (78-fold), respectively, in the *rolD::acdS* under salt stress compared to the wild-type DH55. Csa02g061320 was up-regulated 20-fold in the non-transgenic line by salt stress, again representing significant attenuation of the stress response (Supplemental Data 2).

#### Abscisic acid (ABA)

Genes encoding positive regulators of ABA signalling, namely 9-cis-epoxycarotenoid dioxygenase 3 (NCED; Csa19g021150 and Csa15g018820), which is involved in ABA biosynthesis, and ABA Insensitive 2 (Csa02g061490) were less induced, while that encoding ABA-induced PP2C5 (Csa05g012640) was down-regulated upon salt stress in plants grown in soil treated with *P. migulae* 8R6. The expression of genes encoding negative regulators of ABA signal transduction, including Highly ABA-Induced PP2C protein 1 (three homeologous genes Csa02g064780, Csa18g032370 and Csa11g092890), Highly ABA-Induced PP2C protein 2 (three homeologous genes Csa03g010830, Csa17g010800 and Csa14g008830) and PP2CA (Csa15g015150) were still induced by salt stress, but approximately threefold less in these plants. These genes were induced up to 250-fold in the control line in response to salt stress in the absence of PGPB (Supplemental Data 2).

Contrary to the treatment with *P. migulae* 8R6, expression of *acdS* in *C. sativa* did not greatly affect the expression of genes involved in ABA biosynthesis or signal transduction. Csa04g054460, another homeologue of Csa05g012640 encoding PP2C5, a positive regulator of ABA signal transduction, was less induced (fivefold) compared to the non-transgenic line, but only in the *rolD::acdS* line. Csa14g008830 encodes ABA-induced PP2C, a negative regulator of ABA signaling, and was less induced both in plants grown in soil treated with *P. migulae 8R6* and in the *acdS* transgenic lines. This gene was induced 70-fold in the wild-type DH55 control by salt stress (Supplemental Data 2).

#### Ethylene

As shown above, ACC deaminase reduces ethylene production in plants exposed to salt stress. In accordance, genes encoding Ethylene Responsive Factors (ERF), such as Csa03g031680 and Csa17g039230 encoding ERF11, Csa01g017750 (ERF4), Csa14g063540 and Csa17g093440 encoding ERF8, as well as Csa03g046520 and Csa17g069360 (RAP2.6), which are involved in ethylene-activated signaling, were less induced in vegetative tissue of plants grown in soil treated with *P. migulae* 8R6 compared to the untreated control during salt stress. In the *35S::acdS* line, two other homeologues of Csa01g017750 encoding ERF4 (Csa15g019690 and Csa19g021900), Csa20g081680 (ERF2), as well as Csa11g018190 and Csa10g016710 (ACC synthase 7) were down-regulated or less induced after salt treatment. Similarly, Csa19g033660 (ERF1), Csa03g031680 (ERF11), Csa12g047140 and Csa11g064550 (ethylene responsive element binding factor 1; EBF1), as well as Csa20g081680, Csa18g002590 and Csa14g006260 (EBF2), which are involved in ethylene signaling, were down-regulated or less induced in the *rolD::acdS* line after salt treatment. Many genes involved in ethylene biosynthesis, including Csa14g006260 and Csa17g008270 (ACC oxidase 4), Csa03g001750 and Csa17g001950 (ACC synthase 2), as well as Csa11g018190, Csa12g025860 and Csa10g016710 (ACC synthase 7) were also down-regulated or less induced; these were induced up to 500-fold in wild-type DH55 after salt treatment. In fact, Csa08g022870 (Ethylene and Salt Inducible 3) was the only ethylene-related gene that was induced and only in the *35S::acdS* line (Supplemental Data 2).

#### Jasmonic acid (JA)

Several genes involved in JA biosynthesis were down-regulated in plants grown in soil treated with *P. migulae* 8R6 compared to the untreated control plants after salt treatment, while no genes related to JA biosynthesis or signaling were differentially-expressed in the vegetative tissues of the *acdS* transgenic lines. Genes involved in JA biosynthesis that were down-regulated or less induced in the *P. migulae* 8R6-treated plants included Csa17g023140 and Csa03g021200 (lipoxygenase 3), Csa07g039620 (lipoxygenase 4), Csa19g040810 and Csa01g038310 (oxophytodienoate-reductase 3), as well as Csa06g005320, Csa09g008880 and Csa03g038080 (allene oxide cyclase 1, 2 and 3). Most of these genes were highly induced (up to 26-fold) by salt stress in the untreated control (Supplemental Data 2).

### Protein degradation and cellular turnover

The environmental conditions surrounding plants are constantly changing and post-translational regulation of protein levels (regulatory and functional) is an important mechanism underlying adaptation^32^. The ubiquitination pathway in particular plays a role in altering the plant proteome in response to environmental stresses^33,34^ and many genes involved in protein degradation/turnover were less induced, both in plants treated with *P. migulae* 8R6 and in the *acdS* transgenic lines, compared to the untreated, non-transgenic control during salt stress (Supplemental Data 2) providing evidence for a common mechanism regardless of the means for ACC deaminase delivery. Fully, 25% of the genes commonly down-regulated in the *acdS* transgenic lines were associated with protein degradation.

### Stress response

#### Abiotic stress

Plant stress responses and development are tightly connected processes and their precise regulation is required to maintain fitness when a stress is encountered. Eight genes related to different abiotic stresses were down-regulated or less induced in vegetative tissues of plants grown in soil treated with *P. migulae* 8R6 during salt stress, including two (Csa18g023200 and Csa02g045340) encoding Responsive to Desiccation 29B (RD29B)*.* Furthermore, three genes encoding Responsive to ABA 18 (RAB18; Csa18g042170, Csa11g104590 and Csa02g076390) were also less induced in these plants (Supplemental Data 2). The expression of both *RAB18* and *RD29B* was highly induced 68 and 244-fold, respectively, in vegetative tissues of untreated *C. sativa* upon exposure to salt stress (Supplemental Data 2).

Although Late Embryogenesis Abundant (LEA) proteins are classified under the GO category “development process”, many members of this family play an important role in the abiotic stress response and stress tolerance^35^. Seven genes encoding LEA proteins were less induced and two were down-regulated in plants grown in soil treated with *P. migulae* 8R6 compared to the control (Supplemental Data 2). These were highly up-regulated (up to 185-fold) by salt stress in untreated *C. sativa* suggesting that ACC deaminase has impacted overall stress levels. In the *rolD::acdS* line, three homologous genes encoding LEA5 were less induced (Csa13g054850 and Csa02g005610) or down-regulated (Csa08g052090), as was a gene encoding NDR1/HIN1-like 6 (NHL6; Csa07g029570, less induced) which is also responsive to abiotic stress^36^.

#### Biotic stress

Genes encoding AvrRrt2-Induced Gene1 (Csa03g038080) and Receptor-like protein 22 (Csa16g008730), which are involved in the response to bacterial pathogens, were the only genes related to biotic stress that were differentially-regulated in plants grown in soil treated with *P. migulae* 8R6. However, 11 genes related to biotic stress were less induced or down-regulated in the *35S::acdS* line under salt stress, including Csa16g038950 (Pathogenesis-Related Protein; PR5), Csa11g001970 and Csa10g001780 (Enhanced Disease Susceptibility 5; EDS5) and Csa11g032780 (Suppressor of *npr1*, constitutive 1) which were all less induced compared to the non-transgenic line after salt stress. In the *rolD::acdS* line, 28 genes related to biotic stress were less induced or down-regulated, including those encoding PR1 (Csa01g035420, Csa19g044900 and Csa15g064830), PR5 (Csa16g038950, Csa07g046470 and Csa09g079640), a PR5-like protein (Csa09g079620), and EDS5 (Csa10g001780 and Csa11g001970) (Supplemental Data 2).

### Signalling

#### Transcription factors (TF)

The expression of several genes encoding NAC (NAM, ATAF1/2, CUC2-domain) transcription factors were less induced or down-regulated in plants grown in soil treated with *P. migulae* 8R6 and in the *acdS* transgenic lines compared to the wild-type control during salt treatment. For example, three homoleogous genes (Csa12g023460, Csa11g016750 and Csa10g015440) which encode Responsive to Desiccation 26 (RD26, NAC domain-containing 72) were induced threefold less in *P. migulae* 8R6-treated plants. Genes encoding NAC019 (Csa17g093080, Csa14g063210, and Csa03g060000), NAC055 (Csa01g018090 and Csa19g022270) and NAC102 (Csa18g037380) were less induced in *P. migulae* 8R6-treated plants, as well as in the *rolD::acdS* (NAC019 and NAC055) and *35S::acdS* lines (NAC102). However, all three homeologues encoding NAC019 were less induced in the *P. migulae* 8R6-treated plants, while only the homeologue on sub-genome III (Csa03g060000) was less induced in the *rolD::acdS* line. Genes encoding NAC102 (Csa18g037380), NAC067 (Csa02g004650) and NAC043 (Csa05g002390), were less induced or down-regulated in the *35S::acdS* line. In the *rolD::acdS* line, genes encoding NAC019 (Csa03g060000) and NAC055 (Csa19g022270), and other NAC proteins such as NAC036 (Csa19g053810), NAC042 (Csa05g007460), NAC067 (Csa02g004650), NAC069 (Csa02g004670) and NAC087 (Csa13g021170) were less induced or down-regulated (Supplemental Data 2).

In total, 48 genes encoding other TFs, mostly from the AP2/EREBP, WRKY, MYB and zinc finger (ZF) families, including ERF17 (Csa03g023030), Dehydration Responsive Element-binding Protein 2A (DREB2A; Csa13g007380, Csa20g006670 and Csa08g059070), and AP2.6 (Csa03g046520 and Csa17g069360) were less induced or down-regulated in plants grown in soil treated with *P. migulae* 8R6. All of these genes were highly up-regulated (up to 116-fold) by salt stress in untreated control plants in response to salt. Two homeologous genes (Csa18g029750 and Csa11g090310) and Csa18g024230 encoding a MYB-like TF were the only TF genes that were induced in plants grown in soil treated with *P. migulae* 8R6 (Supplemental Data 2). Of the genes encoding MYB TFs that were less induced in the *P. migulae* 8R6-treated plants under salt stress, some of the more notable ones encoded MYB20 (Csa16g023660), MYB74 (Csa02g009840, Csa08g049290 and Csa13g053050), MYB102 (Csa11g025000, Csa12g036900 and Csa10g021970) and MYB108 (Csa01g007530). In response to salt stress in untreated *C. sativa*, genes encoding MYB20 and MYB108 were up-regulated tenfold, MYB102 up to 40-fold and AtMYB74 up to 1500-fold (Supplemental Data 2).

In total, 16 and 64 genes encoding TFs (other than NAC) were less induced or down-regulated in the *35S::acdS* and *rolD::acdS* transgenic lines, respectively, with most encoding members of the AP2/EREBP, WRKY, MYB, ZF and basic Helix-Loop-Helix (bHLH) families. Among those in common, four genes were less induced, including Csa18g010220 (bHLH 92) which is involved in regulating the circadian clock in *A. thaliana*^37^, or down-regulated, such as Csa02g073920 which encodes WRKY51 and is involved in repression of JA signaling^38^. Over-expression of some genes encoding WRKY TFs increases tolerance against some biotic and abiotic stresses; however, the beneficial effects were often accompanied by some undesirable phenotypes^39^. In the *rolD::acdS* line, genes encoding WRKY25, 30, 31, 33, 38, 47, 50, 51, 53 and 70 were less induced or down-regulated (2 to 40-fold) compared to the wild-type line under salt stress (Supplemental Data 2).

Csa19g025930 encodes a TF with a TCP domain (Branched 1) and was less induced in the *35S::acdS* line. No genes encoding AP2/EREBP TFs were differentially-regulated in the *35S::acdS* line, while two genes encoding bZIP TFs (Csa06g033960 and Csa09g070700) were less down-regulated and one encoding a Homeobox protein (Csa11g094320) were down-regulated. Eleven genes encoding AP2/EREBP TFs were less induced or down-regulated in the *rolD::acdS* line (Supplemental Data 2); these included Csa14g016230 (Regulator of the ATPase of the vascular membrane, RAV1), Csa16g028940 and Csa07g034530 (RAV2 or Tempranillo 2), Csa14g032810 and Csa03g029510 (Tempranillo 1) which were down-regulated and Csa12g027700 (C-Repeat/DRE binding factor 2; CBF2) which was induced fourfold less compared to the non-transgenic line after salt treatment. Four genes encoding B3 or bHLH TFs were induced or less down regulated.

## Discussion

In a previous study, *C. sativa* plants grown in soil amended with PGPB that produce ACC deaminase or lines expressing the bacterial *acdS* gene grew better in a salt mixture formulated to mimic naturally saline soils on the North American prairies^16^. Most other published studies on salinity tolerance use only NaCl, therefore, the studies described herein and a prior study on the root transcriptome^18^ used NaCl at the same EC values as the natural saline compositions. Regardless of the type of salt treatment, *C. sativa* lines expressing *acdS* or plants grown in soil treated with PGPB exhibited less decline in shoot length and weight, and reduced impact on chlorophyll content and photosynthetic yield. PGPB mutants lacking *acdS* also positively impacted certain aspects related to salinity tolerance, such as photosynthetic yield, though to a lesser degree than the wild-type strains, which may be attributed to the ability of these strains to promote plant health by producing plant growth-regulators and increasing the solubility of soil minerals^40^. It is noted here that transcript abundance generally, but does not always, correlate with protein levels. However, this type of study permits examination of a broad suite of pathways and provides clues as to the mechanisms underlying tolerance to salinity stress as discussed below.

### Impact of ACC deaminase on photosynthesis

Salt treatment negatively impacts photosynthesis and the expression of genes related to photosynthesis. ACC deaminase reduced the negative effect of salt on photosynthesis by inducing the expression of genes involved in photosystem I or II and the light harvesting complex, as in plants grown in soil treated with *P. migulae* 8R6, or by reducing the expression of chlorophyllase, as in the *35S::acdS* line. Chlorophyllase is involved in chlorophyll degradation^41^ and reducing its activity improves photosynthetic efficiency and plant productivity under stress conditions^42^. While photosynthetic capacity improved in the *rolD::acdS* line, no significant differences were observed in the expression of photosynthetic genes in the vegetative tissues of this line after salt treatment compared to the non-transgenic line. Interestingly, photosynthetic genes are also expressed in root tissues^12,43^ and ACC deaminase delivered by PGPB or in transgenic *C. sativa* lines attenuates the negative impact of salt on their expression^18^.

This raises the possibility that sensing of salinity stress in roots and subsequent alteration in the expression of photosynthesis-related genes in roots affects photosynthetic capacity in vegetative tissues. The root meristem harbors cells that sense salt stress^44^and this can direct the behavior of other tissues, such as the closure of leaf stomata within seconds after exposure of roots to salt^45^.

### Phytohormones

Ethylene signaling is essential for regulating plant growth and development in response to different environmental stresses and, specifically, is important for acclimation to saline conditions^46,47^. However, elevated levels of ethylene impede plant growth leading to smaller rosettes, earlier flowering and reduced seed production^48^, as well as the onset of senescence, chlorosis and leaf abscission^14^, and a negative feedback mechanism by ethylene has been proposed in salinity stress^49^. In the current study, the level of ethylene was found to be lower in *C. sativa* plants exposed to salt which seemed incongruent with its role in salinity stress; however, it should be noted that we sampled 13 days after the application of salt and very few studies have examined the response to chronic salt exposure^50^. Furthermore, increases in ethylene production associated with salt exposure recover to control levels within 24 h after treatment in salt-tolerant species^51^. *Camelina sativa* is comparatively salt-tolerant and this data supports the notion that ethylene production/sensitivity may already be highly regulated in this species, but that ACC deaminase helps to reduce levels under salt stress even further. Indeed, the expression of many genes involved in ethylene synthesis or ethylene signaling are highly induced in *C. sativa* by salt treatment, for example *ACC synthase 2* and *7* were induced almost 400- and 500-fold, respectively^12^. The negative effect of ACC synthase and ACC oxidase, and resultant ethylene biosynthesis on salt tolerance has been documented^49,52^. The effect of high levels of stress ethylene can be reduced by ACC deaminase application which restores normal plant growth habit while inducing systemic tolerance under stress^14–16,53^. In the current study, not only ethylene, but the expression of genes involved in ethylene synthesis or ethylene signaling was reduced by ACC deaminase application. This indicated that the ability of ACC deaminase to diminish or attenuate the ethylene response goes beyond simply lowering ethylene levels, but also impacts the expression of genes involved in its synthesis and responsiveness.

Comparison of the phenotypes and the expressed genes in the *35S::acdS* and *rolD::acdS* lines indicated that moderate *acdS* expression was more effective than high expression in eliminating the negative effects of ethylene production caused by salt stress. The expression of *acdS* was 60-fold higher in the *35S::acdS* line compared to the *rolD::acdS* line; however, the number of ethylene-related genes that were differentially-expressed (mostly down-regulated or less induced) was three times higher in the *rolD::acdS* line. In addition, the down-regulation or the attenuation of induction was more severe in the *rolD::acdS* line, for example the three homeologous genes encoding ACC synthase 7- were 27-fold less induced in the *rolD::acdS* line, but only five-fold less in the *35S::acdS* line compared to the wild-type line upon salt treatment. Some controversy exists with respect to the role of ethylene in salt stress tolerance in different plants, for example over-expression of genes encoding a number of ERFs enhanced salt tolerance in *A. thaliana*^54^. In the transgenic lines expressing *acdS*, genes involved in ethylene biosynthesis, such as those encoding ACC synthase or ACC oxidase, were less induced compared to the wild-type line after salt treatment. However, in plants grown in soil treated with *P. migulae* 8R6, most of the genes related to ethylene that were less induced encoded negative regulators of ethylene signaling^55^. This seems counter-intuitive as down-regulation of a negative regulator should enhance the effect of ethylene. However, several of these regulators are also involved in crosstalk between ethylene and ABA signaling, such as ERF4, ERF7 and ERF12^55,56^, and, therefore, the actual mechanism and interplay is likely far more complex.

Auxin signalling and cross-talk also appear to have been affected by ACC deaminase as several auxin-responsive genes encoding members of the Small Auxin Up RNA (SAUR) family were up-regulated in the *35S::acdS* and *rolD::acdS* lines. SAUR 76–78 affect ethylene receptor signaling and promote plant growth^57^. Furthermore, in the *rolD::acdS* line and in plants grown in soil treated with *P. migulae 8R6*, several auxin-responsive genes encoding GH3 family proteins and/or IAA-amino acid hydrolases, which regulate auxin homeostasis by conjugating excess auxins to amino acids^58,59^, were less induced. Auxin conjugates compartmentalize auxins to sequester excess IAA or to protect the free acid from peroxidative degradation^58,60^.

The expression of ABA-dependent genes is highly up-regulated (up to 230-fold) in wild type *C. sativa* in response to salt treatment^12^. However, in vegetative tissues of plants grown in soil treated with *P. migulae* 8R6, genes encoding enzymes involved in ABA biosynthesis (NCED) or positive regulators of ABA signal transduction (Insensitive 2 and PP2C5) were less induced, as were several genes encoding negative regulators of ABA signal transduction. This implies that ABA is important for salt tolerance, but that a balance between the positive effect of ABA on stress tolerance and its negative effect on growth and development is necessary. After sensing salinity, two type of reactions (immediate and long-term response) are coordinated by ABA. The immediate response involves inhibition of cell elongation; however, upon prolonged exposure to salt stress, ABA promotes plant growth, but at a lower rate than in the absence of salt stress^44^. The extent of growth recovery depends on the capacity of the plant for acclimation^44,61^. Contrary to the treatment with *P. migulae* 8R6, expression of *acdS* in transgenic *C. sativa* did not greatly affect the expression of genes involved in ABA biosynthesis or signal transduction indicating that ACC deaminase may not be the only reason for the PGPB-mediated effect.

In general, treatment of soil with a *P. migulae* 8R6 strain producing ACC deaminase negatively affected ethylene signaling, auxin and JA biosynthesis and signalling. In addition, ABA production and signalling was also altered in a way that likely reduces the adverse effect of ABA on plant growth and productivity during salt stress. However, PGPB treatment seems to have had a positive effect on the regulation of the GA signaling. In plants expressing *acdS*, the expression of the genes involved in auxin signalling was positively affected, while the expression of genes involved in cytokinin degradation and ethylene biosynthesis was negatively affected. Fine-tuning of ABA signaling may also be occurring.

### Stress response and leaf senescence

Exposure to biotic and/or abiotic stresses redirects plant physiological activity from vegetative growth towards resistance or defense activation. Tuning the regulation of the corresponding genes is crucial to maximizing plant fitness while not unnecessarily compromising plant growth and development^62,63^. For example, accumulation of plant secondary metabolites, such as anthocyanins and flavonoids, which increase salinity resistance in plants, accompanies leaf senescence and reduces plant yield and biomass^31,64^. The overall trend in the transcriptome data showed a reduction in ethylene production and down-regulation of genes involved in the accumulation of stress-related secondary metabolites in the presence of ACC deaminase.

In addition to the impact on secondary metabolism, several genes encoding NAC transcription factors, including RD26, NAC019, NAC055 and NAC102, and genes encoding RD29B and Responsive to ABA 18, were all down-regulated or less induced in plants grown in soil treated with *P. migulae* 8R6 and the transgenic lines during salt stress. These transcription factors regulate the biotic and abiotic stress response and play essential roles in senescence and chlorophyll degradation^65,66^. Salinity and ethylene both induce leaf senescence^67^. *rd29a* and *rd29b* mutant lines display greater root growth, photosynthesis, and water use efficiency under salt stress^68^ and RD26 is a positive regulator of age and dark-induced leaf senescence in addition to its role in desiccation^69^. NAC019 functions downstream of ethylene insensitive 2 (EIN2) and EIN3, ethylene-activated transcription factors necessary for the enhanced salt tolerance^70^; however, NAC019, NAC055 and NAC102 are positive regulators of leaf senescence^66,67,71^. Two genes encoding Yellow-leaf Specific Gene 5 and 9, and a gene encoding ULTRAPETALA1 were also less induced in plants grown in soil treated with *P. miguale 8R6*. The expression of these genes was up-regulated up to 244-fold by salt in vegetative tissues of untreated *C. sativa*. Yellow-leaf Specific Gene 5 and 9 are involved in leaf senescence and ULTRAPETALA1 acts as a negative regulator of shoot, floral meristem size and flower development^72^.

In addition to the lower induction or down-regulation of genes encoding NAC TFs in the *rolD::acdS* line, genes encoding RAV1 (Related to ABI3/VP11), Tempranillo 1 and Tempranillo 2, and several WRKY TFs (WRKY25, 30, 31, 33, 38, 47, 50, 51, 53 and 70) were less induced or down-regulated. The expression of these genes was up-regulated up to 1000-fold by salt stress in wild-type *C. sativa*. RAV1 is a negative regulator of growth and a positive regulator of leaf senescence. *A. thaliana* lines over-expressing *RAV1* exhibit strong growth retardation and a semi-dwarf stature^73^. *TEM1* and *TEM2* delay flowering and induce leaf senescence when over-expressed in *A. thaliana* and seed germination is more inhibited by salt stress^73,74^. Over-expression of some WRKY genes was reported to increase tolerance to some biotic and abiotic stresses; however, the beneficial effects were often accompanied by unwanted phenotypes^39^. WRKY TFs are the second largest family of TFs involved in senescence^75^ with WRKY22, WRKY30, WRKY53, WRKY54, WRKY70, and WRKY75 playing important roles in leaf senescence^76–79^. Genes encoding ZAT7 and ZF protein 1, which are negative regulators of growth under abiotic stress conditions^80^, were also down-regulated or less induced in the *rolD::acdS* line under salt stress.

## Conclusions

The application of ACC deaminase by *P. migulae* 8R6 or in *acdS* transgenic lines caused a reduction in the expression of stress-responsive genes that are highly induced by salt to levels that may lower or indicate a reduced negative effect on the plant. This is in agreement with earlier observations that the impact of salinity stress on growth and productivity was reduced by treatment of soil with *P. migulae* 8R6 or in *acdS* transgenic lines^16^. In many cases, stress adaptation comes at the expense of growth and productivity, therefore, it is necessary for plants to develop resilient systems to optimize the trade-off between short term survival and long-term growth. The physiological and transcriptome data clearly demonstrated that decreasing the concentration of stress ethylene through the application of ACC deaminase can significantly increase the extent of plant growth and productivity under salt stress in *C. sativa*. Expressing the *acdS* gene under the control of different promoters or through the application of endophytic PGPB sometimes affected the expression of different homeologous genes indicating that in polyploid plants studying the effect of stress on gene expression in different sub-genomes is important. This information may be used as a resource for breeders to target the correct homeologue in *C. sativa* to obtain the desired effect on salinity stress. However, approaches, such as the use of ACC deaminase, that regulate a network of genes may be more effective than targeting a single gene to provide stress tolerance and reduce the negative impact on growth and yield production.

## Supplementary Information

Supplementary Information 1.

Supplementary Information 2.

Supplementary Information 3.

Supplementary Information 4.

Supplementary Information 5.

## Data Availability

The RNA-Seq data were deposited to NCBI Gene Expression Omnibus under the accession code GSE132600.
