# Supplementary material for: Gene expression patterns in shoots of Camelina sativa with enhanced salinity tolerance provided by plant growth promoting bacteria producing 1-aminocyclopropane-1-carboxylate deaminase or expression of the corresponding acdS gene
Source: Sci Rep. 2021 Feb 19;11:4260. doi: 10.1038/s41598-021-83629-8 (PMC7895925; doi:10.1038/s41598-021-83629-8)
Supplement: Supplementary file 3 — Supplementary Information 3. [file 41598_2021_83629_MOESM3_ESM.pdf]

Supplemental Figure 1. Expression of select *C. sativa* genes after salt stress (15 dSm-1) as determined by RNA-Seq and droplet digital PCR (dd PCR) analysis. Plots show expression (fold change) in control (wild type DH55) plus salt, *35S::acdS* and *rolD::acdS* transgenic lines or after treatment with the plant growth promoting bacterium *P. migulae* 8R6 relative to the control without salt (set at 1.0) and are reported as means and standard errors of three biological replicates.

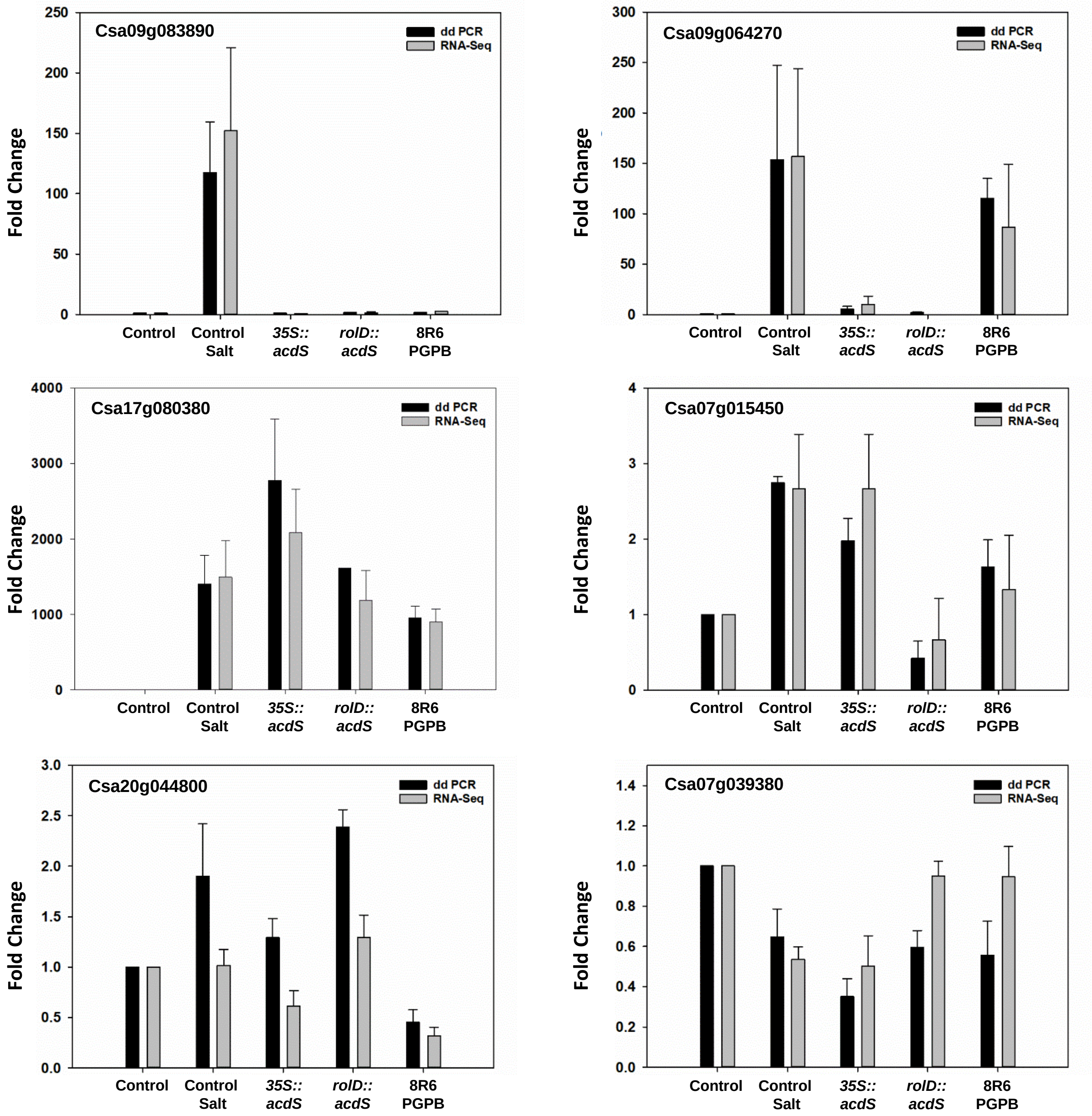

Csa09g083890
Csa09g064270
Csa17g080380
Csa07g015450
Csa20g044800
Csa07g039380
dd PCR
RNA-Seq
Fold Change
Control
Control Salt
35S:: acdS
rolD:: acdS
8R6 PGPB
0
50
100
150
200
250
300
1000
2000
3000
4000
1
2
3
4
0.0
0.5
1.0
1.5
2.0
2.5
3.0
0.2
0.4
0.6
0.8
1.2
1.4
