# Supplementary material for: Gene expression patterns in shoots of Camelina sativa with enhanced salinity tolerance provided by plant growth promoting bacteria producing 1-aminocyclopropane-1-carboxylate deaminase or expression of the corresponding acdS gene
Source: Sci Rep. 2021 Feb 19;11:4260. doi: 10.1038/s41598-021-83629-8 (PMC7895925; doi:10.1038/s41598-021-83629-8)

Supplemental Figure 2. MAPMAN pathways showing categories of genes that were differentially-expressed in *C. sativa* grown in soil treated with *P. migulae* 8R6 or transgenic lines expressing *acdS* under the control of the *35S* or *rolD* promoters relative to the wild type line under salt stress. Output from MAPMAN software version 3.5.1R2 (http://mapman.gabipd.org/).

Regulation

8R6-PGPB

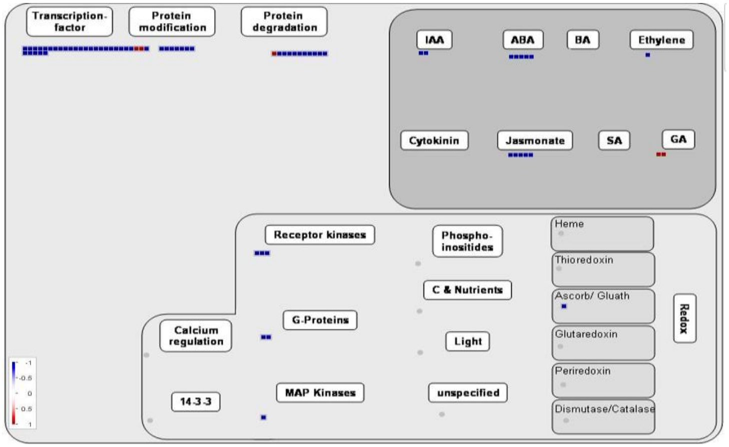

35S::acdS

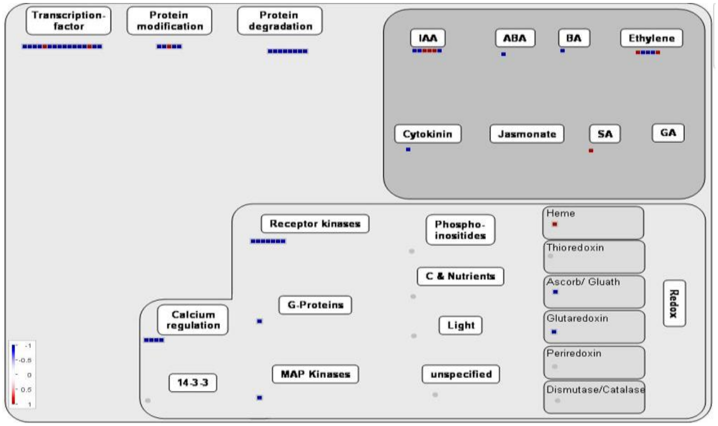

rolD::acdS

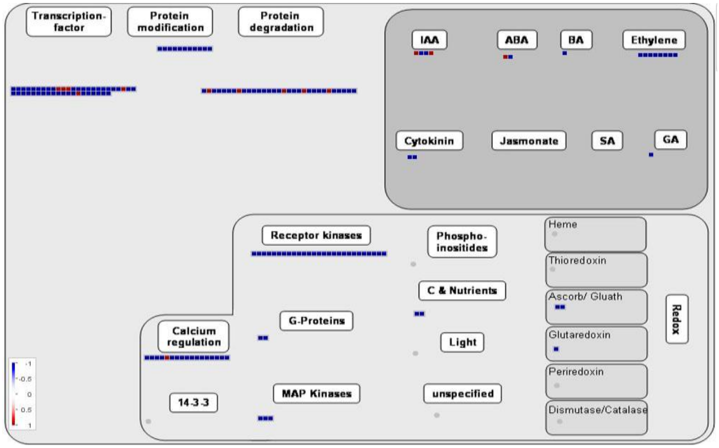

Biotic Stress Response

8R6-PGPB

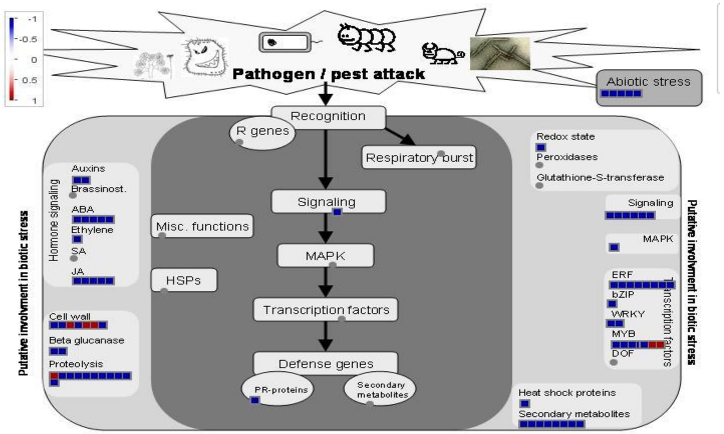

35S::acdS

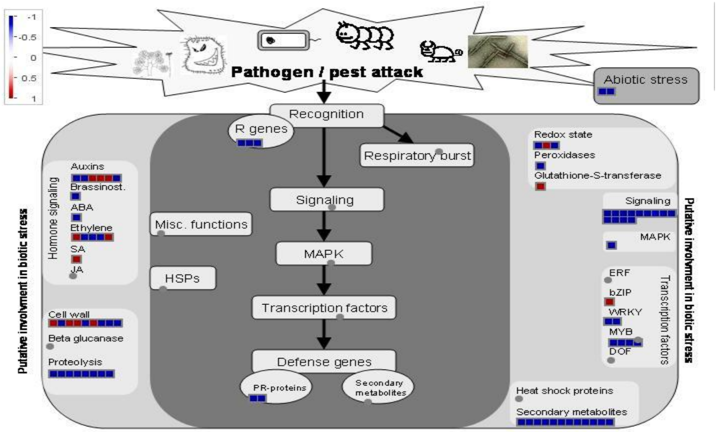

rolD::acdS

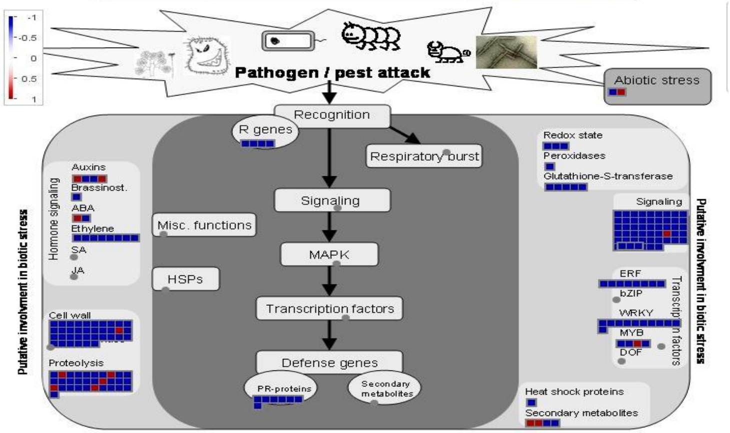

Metabolism

8R6-PGPB

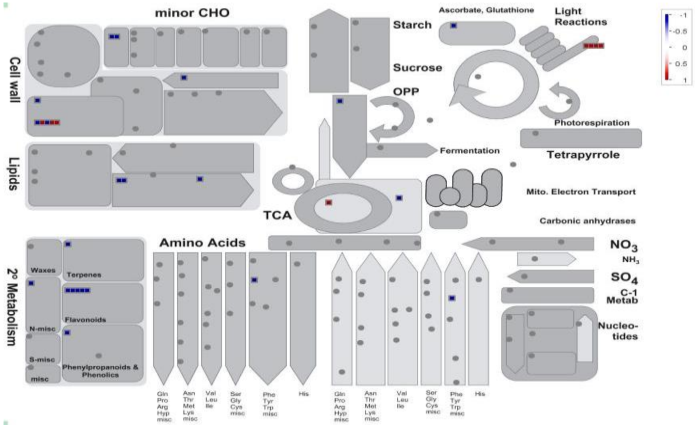

35S::acdS

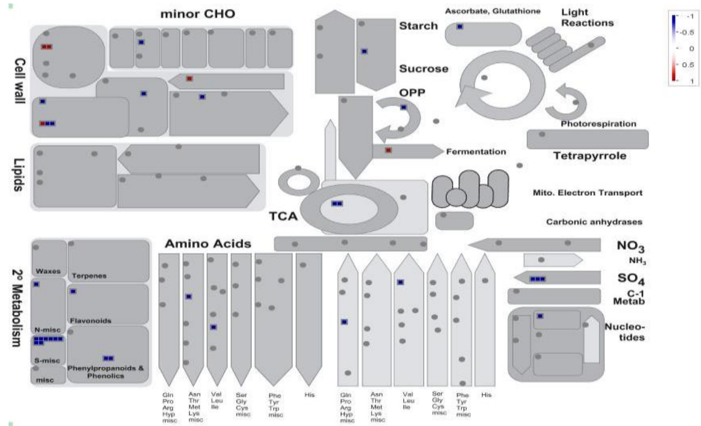

rolD::acdS

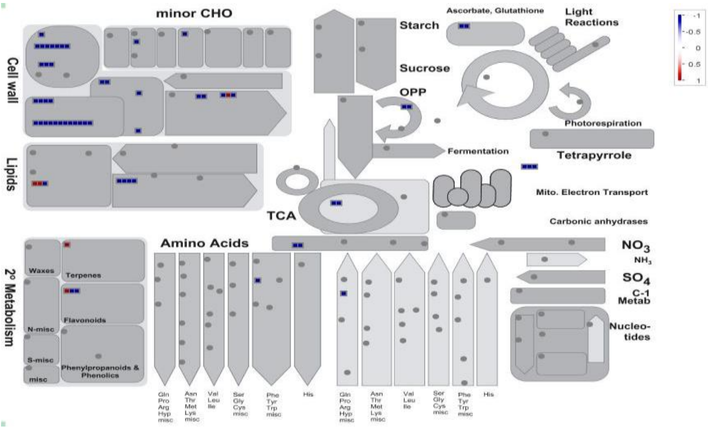

Supplement: Supplementary file 4 — Supplementary Information 4. [file 41598_2021_83629_MOESM4_ESM.pdf]
