# Supplementary material for: Gene expression patterns in shoots of Camelina sativa with enhanced salinity tolerance provided by plant growth promoting bacteria producing 1-aminocyclopropane-1-carboxylate deaminase or expression of the corresponding acdS gene
Source: Sci Rep. 2021 Feb 19;11:4260. doi: 10.1038/s41598-021-83629-8 (PMC7895925; doi:10.1038/s41598-021-83629-8)
Supplement: Supplementary file 5 — Supplementary Information 5. [file 41598_2021_83629_MOESM5_ESM.pdf]

Supplemental Figure 3.

Cluster analysis of common differentially-expression genes in vegetative tissue from *C. sativa* lines expressing *acdS* under the control of the *rolD* promoter or the constitutive *CaMV 35S* promoter, or from plants grown in soil treated with *P. migulae 8R6* in the presence of salt (NaCl, 15 dSm^{-1}). Cluster analysis done using JMP software version 15.

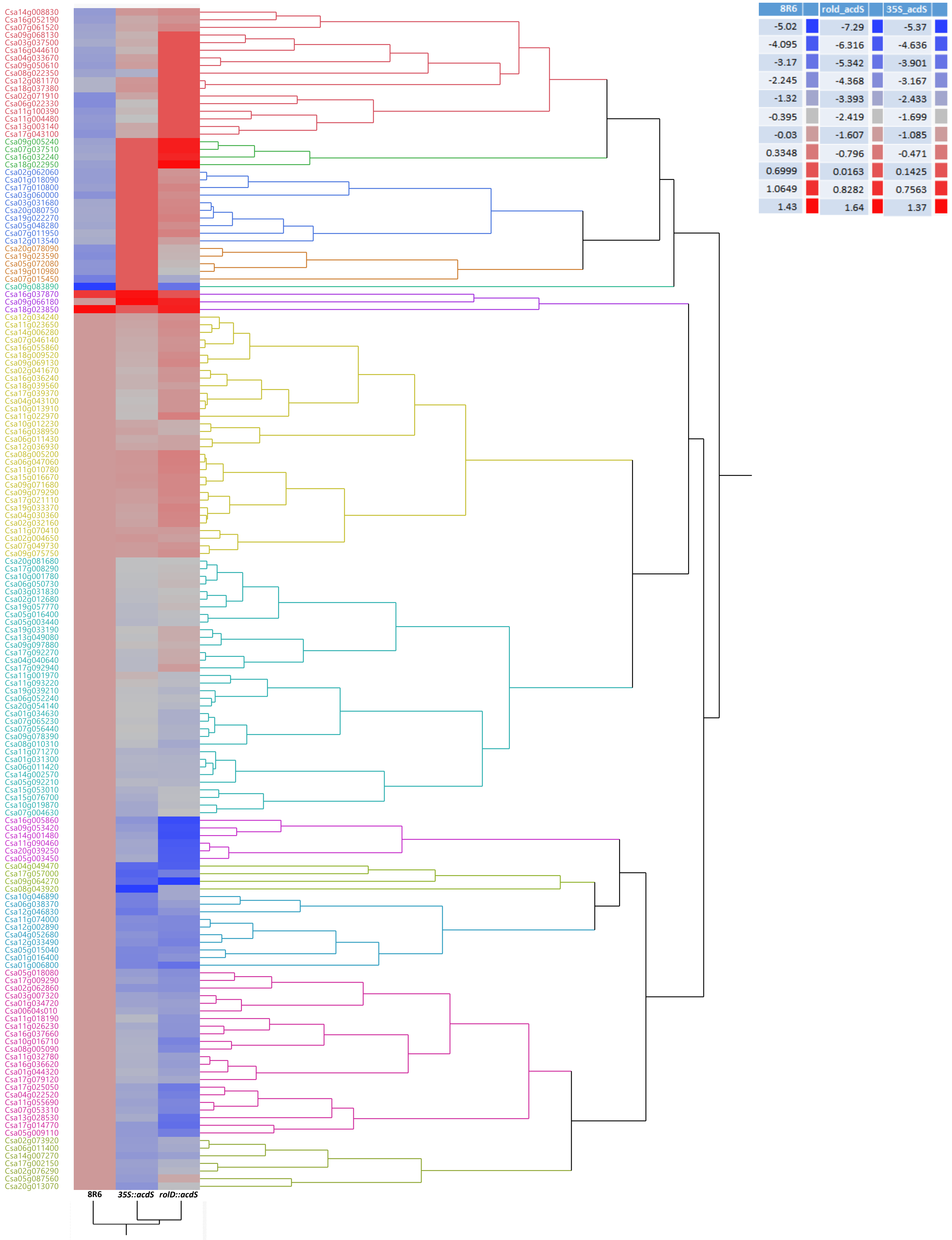
Csa14g008830
Csa16g052190
Csa07g061520
Csa09g068130
Csa03g037500
Csa16g044610
Csa04g033670
Csa09g050610
Csa08g022350
Csa12g081170
Csa18g037380
Csa02g071910
Csa06g022330
Csa11g100390
Csa11g004480
Csa13g003140
Csa17g043100
Csa09g005240
Csa07g037510
Csa16g032240
Csa18g022950
Csa02g062060
Csa01g018090
Csa17g010800
Csa03g060000
Csa03g031680
Csa20g080750
Csa19g022270
Csa05g048280
Csa07g011950
Csa12g013540
Csa20g078090
Csa19g023590
Csa05g072080
Csa19g010980
Csa07g015450
Csa09g083890
Csa16g037870
Csa09g066180
Csa18g023850
Csa12g034240
Csa11g023650
Csa14g006280
Csa07g046140
Csa16g055860
Csa18g009520
Csa09g069130
Csa02g041670
Csa16g036240
Csa18g039560
Csa17g039370
Csa04g043100
Csa10g013910
Csa11g022970
Csa10g012230
Csa16g038950
Csa06g011430
Csa12g036930
Csa08g005200
Csa06g047060
Csa11g010780
Csa15g016670
Csa09g071680
Csa09g079290
Csa17g021110
Csa19g033370
Csa04g030360
Csa02g032160
Csa11g070410
Csa02g004650
Csa07g049730
Csa09g075750
Csa20g081680
Csa17g008290
Csa10g001780
Csa06g050730
Csa03g031830
Csa02g012680
Csa19g057770
Csa05g016400
Csa05g003440
Csa19g033190
Csa13g049080
Csa09g097880
Csa17g092270
Csa04g040640
Csa17g092940
Csa11g001970
Csa11g093220
Csa19g039210
Csa06g052240
Csa20g054140
Csa01g034630
Csa07g065230
Csa07g056440
Csa09g078390
Csa08g010310
Csa11g071270
Csa01g031300
Csa06g011420
Csa14g002570
Csa05g092210
Csa15g053010
Csa15g076700
Csa10g019870
Csa07g004630
Csa16g005860
Csa09g053420
Csa14g001480
Csa11g090460
Csa20g039250
Csa05g003450
Csa04g049470
Csa17g057000
Csa09g064270
Csa08g043920
Csa10g046890
Csa06g038370
Csa12g046830
Csa11g074000
Csa12g002890
Csa04g052680
Csa12g033490
Csa05g015040
Csa01g016400
Csa01g006800
Csa05g018080
Csa17g009290
Csa02g062860
Csa03g007320
Csa01g034720
Csa00604s010
Csa11g018190
Csa11g026230
Csa16g037660
Csa10g016710
Csa08g005090
Csa11g032780
Csa16g036620
Csa01g044320
Csa17g079120
Csa17g025050
Csa04g022520
Csa11g055690
Csa07g053310
Csa13g028530
Csa17g014770
Csa05g009110
Csa02g073920
Csa06g011400
Csa14g007270
Csa17g002150
Csa02g076290
Csa05g087560
Csa20g013070
8R6
35S::acdS
rolD::acdS
8R6
rold_acdS
35S_acdS
-5.02 -7.29 -5.37
-4.095 -6.316 -4.636
-3.17 -5.342 -3.901
-2.245 -4.368 -3.167
-1.32 -3.393 -2.433
-0.395 -2.419 -1.699
-0.03 -1.607 -1.085
0.3348 -0.796 -0.471
0.6999 0.0163 0.1425
1.0649 0.8282 0.7563
1.43 1.64 1.37
